# Pharmacokinetic study of single- and multiple-dosing with metolazone tablets in healthy Chinese population

**DOI:** 10.1186/s40360-017-0178-x

**Published:** 2017-11-16

**Authors:** Xueqing Li, Rutao Wang, Yang Liu, Yun Liu, Heng Zheng, Yabo Feng, Na Zhao, Hongbin Geng, Wanzhi Zhang, Aidong Wen

**Affiliations:** 1 Department of Pharmacy, Xijing Hospital, Fourth Military Medical University, Xi’an, China; 2 Xi’an Libang Zhaoxin Biological Technology Co., Ltd, Xi’an, China; 3 Eliving Pharmaceutical Co., Ltd, Shenyang, China; 4 Department of Pharmacy, Tongji Hospital, Wuhan, China

**Keywords:** Metolazone, Pharmacokinetics, Food, Gender, Tolerability, Clinical trial, LC-ms/ms

## Abstract

**Background:**

Metolazone is a diuretic, saluretic and antihypertensive chemical compound from the quinazoline category that possesses medicinal features similar to those of other thiazide diuretic drugs. However, the pharmacokinetics of metolazone in the Chinese population has rarely been studied.

This study aimed to examine the pharmacokinetic characteristics, safety characteristic, and tolerability of metolazone in healthy Chinese subjects after single and multiple doses taken orally as well as the effects that food and gender have on oral metolazone pharmacokinetic parameters.

**Methods:**

An open-label, randomized, and single- and multiple-dosing investigation was performed in healthy Chinese subjects. The investigation included 3 study groups: the 0.5 mg, 1 mg and 2 mg dose groups were the single-dose study groups in the first stage. Eligible volunteers were randomly and orally administered a single 0.5 mg, 1 mg, or 2 mg metolazone tablet. The 0.5 mg dose group was also part of the multiple-dose study group, and the 1 mg dose group was the food-effect study group in the second stage. Human plasma samples were gathered pre-dosing and up to 48 h after dosing. The human plasma sample concentration of metolazone was quantified using a validated liquid chromatography tandem mass spectrometry method. Pharmacokinetic data were calculated by a noncompartmental analysis method using WinNonlin version 6.4. Tolerability was evaluated based on adverse events, medical examination, 12-lead ECG, and other clinical laboratory exams.

**Results:**

Thirty eligible subjects (15 men and 15 women) were registered in our investigation and completed all of the study stages. The AUC and C_max_ showed dose proportionality after a single dose based on the linear-regression analysis. A comparison of the pharmacokinetic data revealed that the differences between the male and female groups were not statistically significant. The t_max_ of metolazone was increased by approximately 100% in the fed condition. Metolazone was well tolerated at the tested dose, and no adverse effects were observed.

**Conclusions:**

Single dosing with 0.5 mg, 1 mg, or 2 mg metolazone yielded linear plasma pharmacokinetic properties in healthy Chinese subjects. Multiple oral doses of metolazone did not display significantly different distributions or elimination characteristics from those observed for a single dose. Gender factors did not appear to influence the pharmacokinetic parameter variation of metolazone. The t_max_ of metolazone increased in the fed condition. Metolazone was well tolerated at the tested dose in this study.

**Trial registration:**

This investigation is retrospectively registered at chictr.org.cn (ChiCTR-IIR-17012929, October 09 2017).

## Background

Metolazone(2-methyl-3-o-tolyl-6-sulphamyl-7-chloro-1, 2,3,4-tetrahydro-4-quinazolinone) is a diuretic, saluretic and antihypertensive chemical compound that belongs to the quinazoline category. It has medicinal features similar to those of other thiazide diuretic drugs and is an inhibitor of sodium potassium excretion [1–5].

Metolazone pharmacokinetics data are obtainable from previous studies on Chinese volunteers, including male and female volunteers and those participating in multiple oral dose administrations [1–5]. However, there are no data on the effects that food and gender have on this drug in Chinese volunteers. Therefore, this investigation was conducted to investigate the pharmacokinetic characteristics of metolazone in the Chinese population. It was a registered study permitted by the China Food and Drug Administration (approval No. 2005 L04730) before our clinical trial.

According to a previously reported study, single doses of 0.5 mg, 1 mg and 2 mg metolazone are rapidly and completely absorbed, with the peak plasma concentrations attained approximately 1.5 h following administration. The mean elimination half-life of metolazone has been observed to be between 6 and 8 h, and the area under the curve is reported to range from 30 ng•h/ml to 160 ng•h/ml [6].

Our study is a phase I clinical study of metolazone in Chinese volunteers. Our investigation assessed the safety character, tolerability, and pharmacokinetic parameters of single (0.5 mg, 1 mg and 2 mg) and multiple (0.5 mg) oral doses of metolazone. This study is the first to evaluate the role that food and gender have on this drug in Chinese volunteers. Few studies examining metolazone have been reported in the literature, and our investigation provides pharmacokinetic information for future reference.

## Methods

### Inclusion and exclusion criteria

Our inclusion criteria included healthy male and female Chinese subjects who were 18 to 45 years of age. The body mass index of qualified volunteers was between 18 and 24. Other inclusion criteria comprised the physical status diagnosed by health and family history, a medical examination, 12-lead ECG values, laboratory exams (e.g., haematology, blood biochemistry, hepatic function, urinalysis, hepatitis B surface antigen, and exams for alcohol and other substance abuse) and smoking habits.

Our exclusion criteria included allergies or cardiopathic disease history, pulmonary, renal, hepatic, gastrointestinal, haematologic abnormalities, and severe or long-lasting disease. Additional exclusion criteria included participating in any other clinical study, having taken drugs within the previous 30 days, pregnant or lactating women, and women who were not taking effective precautions to avoid pregnancy.

### Ethics

The protocol, informed consent, and related documents of this investigation were reviewed by the Independent Ethics Committee of Tongji Hospital, Huazhong University of Science and Technology (Wuhan, China). The entire study process was managed in compliance with the Declaration of Helsinki regarding medical ethics and the guiding principles of the International Conference on Harmonization Guidelines for Good Clinical Practice. All volunteers were required to provide informed consent before being screened for enrolment.

### Study design and treatments

**Fig. 1:**
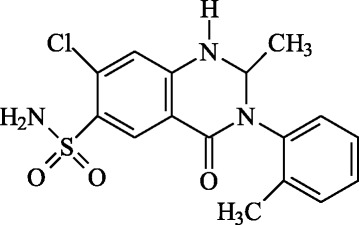
chemical structures of metolazone

Administration of the research drug and human sample collection were completed in the Phase I Study laboratory of the Tongji Hospital clinical trial center, which was under direct medical management by the Huazhong University of Science and Technology. The studied metolazone tablets (the structures are shown in Fig. 1, batch No. 20140114) were provided by Libang Pharmaceuticals Co., Ltd. (Xian, China).

The study enrolled 30 (15 male and 15 female) healthy Chinese subjects. Based on a computer-generated table of random numbers, 30 volunteers were assigned in a 1:1:1 proportion to randomly take a single 0.5 mg, 1 mg, or 2 mg metolazone oral tablet.

This study was designed as an open-label, randomized, single-centre, single- and multiple-dose pharmacokinetic study that included 3 study groups. Groups 1–3 were single-dose study groups in the first stage; qualified volunteers were randomly administered a single 0.5 mg, 1 mg, or 2 mg metolazone tablet. The 0.5 mg dose group was the multiple-dose study group in the second stage, and the 1 mg dose group was the food effects study group in the second stage.

All volunteers arrived at the Phase I Study laboratory 1 day ahead of the drug administration. They were instructed to avoid eating overnight (10 h); then, they were provided standard food after taking a single oral dose (either a 0.5 mg, 1 mg, or 2 mg metolazone tablet) on the dose day in every single oral dose study period. The metolazone tablets were taken with 250 mL of water. More drinking water was allowed 2 h after the dose. Then, the volunteers were provided a unified food (924 kcal; 18% protein, 49% carbohydrate, and 33% fat) 4 and 10 h after the single oral dose administration. Human plasma samples (3 mL) were collected through the indwelling catheter, which was placed in a forearm vein before and at 0.5, 0.75, 1, 1.25, 1.5, 2, 2.5, 3, 4, 6, 8, 10, 12, 24, 36 and 48 h after dosing.

In our multiple oral dose administration stage, the volunteers administered a 0.5 mg metolazone tablet once each day for 6 days. Human plasma samples (3 mL) were taken before dosing on the third, fourth, fifth, sixth, seventh, eighth, and ninth days. On the last day of the multiple oral dose, human plasma samples (3 mL) were gathered before and at 0.5, 0.75, 1, 1.25, 1.5, 2, 2.5, 3, 4, 6, 8, 10, 12, 24, 36 and 48 h after dosing, and this phase was followed by a single oral dose.

The food effect study was conducted on the 1 mg dose group. There was a single oral dose 2-period, 2-sequence crossover study to assess the influence of food on the pharmacokinetic parameters of metolazone tablets in 10 Chinese volunteers. In each administration period, a 1 mg metolazone tablet was orally administered to volunteers under the following fasting or food (high-fat breakfast) situations: (1) after an all-night (at least 10 h) empty stomach or (2) after an all-night, 10-h empty stomach followed by a high-fat caloric meal. The washout period between the fast and fed states was 7 days. The high-fat breakfast was 924 cal, with fat comprising 59% of the total caloric content.

Human plasma samples (3 mL) were gathered through the indwelling catheter, which was placed in a forearm vein before and at 0.5, 0.75, 1, 1.25, 1.5, 2, 2.5, 3, 4, 6, 8, 10, 12, 24, 36 and 48 h after dosing.

The subjects were prohibited from using other drugs, including non-prescription drugs, for 14 days before and during the investigation process. They were prohibited from drinking alcohol, smoking, severe physical activity and caffeine-containing drinks throughout the investigation period.

The biological samples were transferred into plastic tubes, heparin sodium was added to the tubes as an anticoagulant before blood sample collection, and the tubes were instantaneously centrifuged at 3500 rpm. The centrifugation time and temperature were 10 min and 4°C, respectively. The supernatant liquid was drawn into a polypropylene tube and then stored at −70°C until drug determination.

### Metolazone assays

Human plasma concentrations of metolazone were determined via a liquid chromatography-tandem mass spectrometry (LC-MS/MS) method established and validated previous to our clinical trial [7–11]. The determination of the metolazone concentration in the human plasma samples was completed at the Research Center for Drug Metabolism of Tongji University. Chromatography was conducted with an Agilent 1200 HPLC connected to a 6410 Triple Quadrupole mass spectrometer (Agilent Technologies, Palo Alto, CA, USA) equipped with an electrospray source operating in positive ionization mode and a Thermo AQUASIL C18 column (50 mm × 2.1 mm; 5 μm), and the column temperature was sustained at 30°C. The mobile phase consisted of methanol with 0.1% formic acid in water (10:90, *v*/v) and was set at a flow rate of 0.5 mL/min. Human plasma samples were kept frozen at −70°C and protected from light. Plasma samples were spiked with an internal standard and then extracted by partitioning with ethyl acetate. The determination was conducted by triple quadrupole mass spectrometry to monitor the precursor-to-product ion pairs m/z 366.1–259.0 and m/z 306.1–236.1 for metolazone and the internal standard, respectively. The mean accuracy ranged from 91.6% to 106.1%, and the precision of the analysis ranged from 5.7% to 19.0%. The study was analysed for metolazone concentrations by means of an LC-MS/MS method with a concentration range from 0.0198 to 79.0400 ng/mL.

### Pharmacokinetic parameters and statistical analysis

The pharmacokinetic data were summarized using a noncompartmental analysis method calculated using WinNonlin version 6.4 (Pharsight Corporation, Mountain View, CA, USA). C_max_ and t_max_ were calculated from the human plasma concentration time characters for metolazone. The AUC_0-t_ was obtained based on the linear trapezoidal method. The summation of AUC_0-t_ and Ct/λ was applied to calculate the value of AUC_0-t._ The value of Ct was the last measured concentration of human plasma.

To obtain the slope as the value of λ, the log-transformed concentration-time curve was used to conduct linear regression. The ratio of 0.693/λ was applied as the t_1/2_ value. SAS version 9.2 (SAS Institute, Inc., Cary, NC, USA) was used for all statistical analyses in this study. Independent-sample t tests or nonparametric tests were applied to measure statistically significant differences between the pharmacokinetic data. A linear-regression model was adopted to measure the dose proportionality.

The dose proportionality of metolazone over the dose range from 0.5 to 2 mg was estimated through matching an appropriate model. It was assumed that a linear relationship existed between the natural log transformed pharmacokinetic parameter (AUClast, AUCinf, and Cmax) and the natural log transformed dose in the model. The proportionality constant and its corresponding 90% confidence interval (CI) were contrasted with the modified acceptance range. For all analytical results, *P* < 0.05 was considered statistically significant.

### Tolerability assessment

The tolerability assessments consisted of recording and monitoring all adverse events. The volunteers were arranged physical examinations continuously in the Phase I Study laboratory throughout the study. All adverse events were observed and recorded by trained study nurses or researchers. The vital signs were examined throughout the study as follows: pre-dosing and 0.5, 1, 2, 4 and 24 h post-dosing on each dosing day. The medical examinations were assessed based on the screening, pre-dosing, and 24-h post-dosing on every dose day. All abnormal data of the volunteers were documented on the case-report form throughout the study. The laboratory tests, including the 12-lead ECG, haematology, blood biochemistry, hepatic function and urinalysis, were termly examined throughout the study. All laboratory examinations were conducted at the relevant laboratory at Tongji Hospital, Huazhong University of Science and Technology.

### Safety analysis

The safety evaluation of this study was examined at screening and baseline and throughout the study using medical examinations, most important signs (systolic and diastolic blood pressure, pulse rate, respiratory rate, and temperature), 12-lead ECGs values, laboratory examinations (serum chemical analysis, haematologic examining, including coagulation parameters, and urinalysis) and adverse event judgement.

## Results

### Pharmacokinetic parameters

Thirty volunteers (15 men and 15 women) were registered in our investigation, with a mean age (CV) of 21 (9.5%) years (range = 18–25 years), weight of 58.0 (10.2%) kg (range = 50.0–75.0 kg), and height of 167.6 (4.6%) cm (range = 155.0–181.0 cm). All of the participants completed the research. Twenty subjects’ pharmacokinetics parameters were summarized for the 1 mg and 2 mg single oral dose study, and 10 volunteers’ pharmacokinetic parameters were summarized for the 0.5 mg single oral dose and multiple oral dose study.

**Fig. 2:**
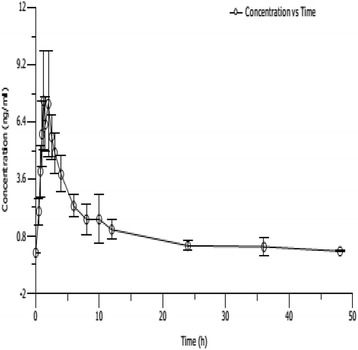
Mean plasma concentrations (mean ± SD) of a single 0.5 mg oral metolazone tablet (*n* = 10)

**Fig. 3:**
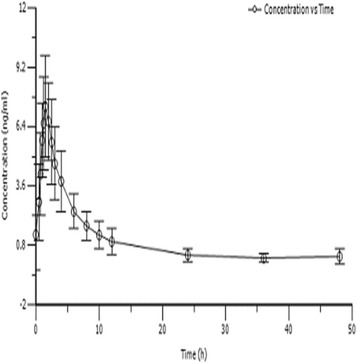
Mean plasma concentrations (mean ± SD) of multiple 0.5 mg oral metolazone tablets (n = 10)

**Fig. 4:**
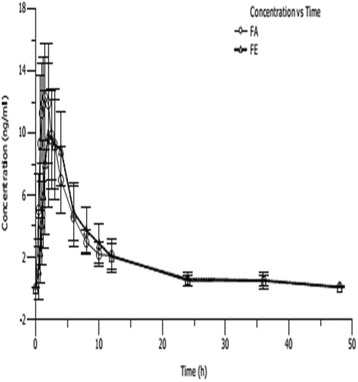
Mean plasma concentrations (mean ± SD) of single-dose 1 mg oral metolazone under fasted and fed conditions (n = 10)

**Fig. 5:**
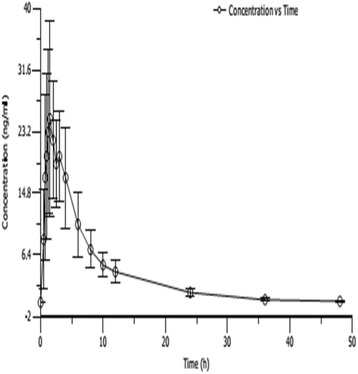
Mean plasma concentrations of a single 2 mg oral metolazone tablet (n = 10)

The mean human plasma metolazone concentration-time curves are illustrated in Figs. 2, 3, 4 and 5. The mean plasma concentrations for the single 0.5 mg oral metolazone tablet are illustrated in Fig. 2. The mean plasma concentrations for the multiple 0.5 mg oral metolazone tablet are illustrated in Fig. 3. The mean plasma concentrations for the single 1 mg oral metolazone tablet under fasted and fed conditions are illustrated in Fig. 4. The mean plasma concentrations for the single 2 mg oral metolazone tablet are illustrated in Fig. 5. The important pharmacokinetic (PK) parameters for metolazone after the single oral dose taken of the 0.5 mg, 1 mg, and 2 mg metolazone tablets and multiple oral dose taken of 0.5 mg metolazone tablets in healthy Chinese subjects are presented in Table 1.

**Table 1:** Primary pharmacokinetic (PK) parameters of metolazone after single-dose administration of 0.5 mg, 1 mg, and 2 mg metolazone tablets and multiple-dose administration of 0.5 mg metolazone tablets in healthy Chinese volunteers

	Single dose			Multiple dose
PK parameter	0.5 mg(n = 10)	1 mg(n = 10)	2 mg(n = 10)	0.5 mg(n = 10)
Kel (1/h)	0.1002 ± 0.0309	0.0966 ± 0.0123	0.0969 ± 0.0181	0.0957 ± 0.0227
C_max_(ng/mL)	8.2252 ± 2.7712	13.8666 ± 4.3227	28.8775 ± 11.3863	8.1967 ± 2.0941
t_max_(h)	1.63 ± 0.46	1.43 ± 0.39	2.05 ± 1.11	1.63 ± 0.41
t_1/2_(h)	7.47 ± 2.06	7.28 ± 0.93	7.42 ± 1.60	7.61 ± 1.76
AUC_0-t_(ng/mL/h)	49.68 ± 14.16	91.31 ± 30.39	184.84 ± 52.39	51.35 ± 15.02
AUC_0-∞_(ng/mL/h)	50.51 ± 14.60	92.41 ± 31.38	186.58 ± 52.50	52.26 ± 15.28
Vd (ml)	108,732 ± 21,357	120,081 ± 23,585	126,326 ± 53,382	122,125 ± 38,729
CL(ml/h)	10,645.1 ± 3021.0	11,755.6 ± 3252.2	11,630.7 ± 3726.0	11,267.2 ± 2770.6
C_avg_(ng/mL)	\	\	\	1.9595 ± 0.5118
C_min_(ng/mL)	\	\	\	0.3962 ± 0.2258
AUCss(ng/mL/h)	\	\	\	1.9595 ± 0.5118

All values are mean (±SD)

*Kel* Rate constant for elimination, *C*_*max*_ Maximum plasma concentration, *t*_*max*_ Time to maximum plasma concentration, *t*_*1/2*_ Apparent plasma terminal elimination half-life, *AUC*_*0-t*_ AUC from 0 h to time t, *AUC*_*0-∞*_ AUC from time 0 extrapolated to infinity, *Vd* Volume of distribution during terminal elimination phase, *CL* Apparent clearance, *Cavg* Average plasma concentration, *C*_*min*_ Minimum observed concentration, *AUC*_*ss*_ AUC of distribution at steady state

All pharmacokinetic parameters are shown using the resultant mean (SD) values. The mean (SD) pharmacokinetic data are listed in Table 1. The value of AUC and C_max_ displayed dose proportionality after a single dose, according to the linear-regression analysis. We performed a linear regression analysis of C_max_, AUC_0-t_ and AUC_0-∞_. The results suggest that a single oral dose of 0.5 mg, 1 mg and 2 mg metolazone appeared to linear plasma pharmacokinetic parameters. The C_max_ for the 0.5 mg to 2 mg dose increased from 8.22 to 28.88 ng/mL, respectively, and the corresponding AUC_0-t_ (from 49.68 to 184.84 ng/mL/h) and AUC_0-∞_ (from 50.51 to 186.58 ng/mL/h) pharmacokinetic values displayed significantly linear trends (Figs. 2, 3, 4, and 5 and Table 1).

Metolazone was rapidly absorbed at the low 0.5 and 1 mg doses, although the absorption slowed at the higher 2 mg dose. The t_max_ was 1.63 and 1.43 h at 0.5 and 1 mg, respectively, and was 2.05 h when the dose reached 2 mg (Figs. 2, 3, 4, and 5 and Table 1).

The mean half-life at the different doses was 7.3 h; there was no obvious difference among the 3 dose groups. The t_1/2_ values were unchanged, even after increasing the dose (7.47 h at 0.5 mg and 7.42 h at 2 mg, respectively) (Figs. 2 and 5, Tables 2 and 4).

**Table 2:** Comparison of pharmacokinetic (PK) parameters in male and female subjects after single-dose administration of 0.5 mg tablets in healthy Chinese volunteers

PK parameter	male(*n* = 5)		female(*n* = 5)		all(*n* = 10)	
	Mean	SD	Mean	SD	Mean	SD
Kel(1/h)	0.0865	0.0257	0.1139	0.0319	0.1002	0.0309
t_1/2_(h)	8.51	2.11	6.43	1.55	7.47	2.06
t_max_(h)	1.60	0.38	1.65	0.58	1.63	0.46
C_max_(ng/ml)	8.3722	3.0768	8.0782	2.7853	8.2252	2.7712
AUC_0-t_(ng·h/ml)	51.06	17.02	48.29	12.53	49.68	14.16
AUC_0-∞_(ng·h/ml)	52.19	17.71	48.82	12.60	50.51	14.60
Vd(ml)	120,906	15,832	96,557	20,127	108,732	21,357
CL(ml/h)	10,574.6	3879.5	10,715.6	2339.0	10,645.1	3021.0
MRT_0-t_(h)	10.08	4.55	7.37	1.49	8.73	3.49
MRT_0-∞_(h)	11.00	4.62	7.85	1.45	9.43	3.63

All values are mean (±SD)

*Kel* Rate constant for elimination, *t*_*1/2*_ Apparent plasma terminal elimination half-life, *t*_*max*_ Time to maximum plasma concentration, *C*_*max*_ Maximum plasma concentration, *AUC*_*0-t*_ AUC from 0 h to time t, *AUC*_*0-∞*_ AUC from time 0 extrapolated to infinity, *Vd* Volume of distribution during terminal elimination phase, *CL* Apparent clearance, *MRT*_*0-t*_ The average residence time of zero time to t, *MRT*_*0-∞*_ The average residence time of zero time to infinite time

The mean human plasma concentration–time characters after a multiple oral dose are illustrated in Fig. 3. The mean (SD) pharmacokinetic parameters are listed in Table 1.

Through repeated administrations of metolazone, the mean human plasma concentrations reached the pre-dosing levels on Days 3 and 9 of the multiple oral dose, indicating that a steady state human plasma metolazone concentration was reached close to Day 6 during the multiple oral dose. The C_min_ and C_avg_ of metolazone were 0.3962 ng/mL and 1.9595 ng/mL, respectively (Fig. 3 and Tables 1 and 5).

In contrast to the single oral dose, the elimination half-life after the multiple oral dose was slightly advanced and decreased to 7.61 h (Table 1). The mean apparent distribution volume was 122,125 L, and the mean human plasma clearance was 11,267 L/h after the orally administered multiple dose; these two parameters were slightly changed compared with those after the single oral dose (*P* > 0.05) (Table 1). Neither the C_max_ nor AUC_0-t_ increased after the multiple dose administration. The accumulation index value of metolazone, which was calculated from AUC single-dose/AUC multiple-dose, was 0.98 (0.28), suggesting no metolazone accumulation. Tables 2, 3, 4 and 5) shows the main pharmacokinetic data between the male and female volunteers and does not identify a major change between the groups, indicating that gender does not influence the pharmacokinetic characteristics of metolazone.

**Table 3:** Comparison of pharmacokinetic (PK) parameters in male and female subjects after single-dose administration of 1 mg tablets in healthy Chinese volunteers

PK parameter	male(n = 5)		female(n = 5)		all(n = 10)	
	Mean	SD	Mean	SD	Mean	SD
Kel(1/h)	0.1000	0.0145	0.0932	0.0100	0.0966	0.0123
t_1/2_(h)	7.05	1.02	7.51	0.87	7.28	0.93
t_max_(h)	1.45	0.37	1.40	0.45	1.43	0.39
C_max_(ng/ml)	11.6412	1.9780	16.0920	5.0744	13.8666	4.3227
AUC_0-t_(ng·h/ml)	78.99	23.89	103.63	33.57	91.31	30.39
AUC_0-∞_(ng·h/ml)	79.81	24.60	105.01	34.84	92.41	31.38
Vd(ml)	132,420	23,825	107,742	17,417	120,081	23,585
CL(ml/h)	13,349.6	3401.1	10,161.6	2424.7	11,755.6	3252.2
MRT_0-t_(h)	8.78	1.32	8.82	1.81	8.80	1.50
MRT_0-∞_(h)	9.23	1.60	9.39	2.11	9.31	1.77

All values are mean (±SD)

*Kel* Rate constant for elimination, *t*_*1/2*_ Apparent plasma terminal elimination half-life, *t*_*max*_ Time to maximum plasma concentration, *C*_*max*_ Maximum plasma concentration, *AUC*_*0-t*_ AUC from 0 h to time t, *AUC*_*0-∞*_ AUC from time 0 extrapolated to infinity, *Vd* Volume of distribution during terminal elimination phase, *CL* Apparent clearance, *MRT*_*0-t*_ The average residence time of zero time to t, *MRT*_*0-∞*_ The average residence time of zero time to infinite time

**Table 4:** Comparison of pharmacokinetic (PK) parameters in male and female subjects after single-dose administration of 2 mg tablets in healthy Chinese volunteers

PK parameter	male(n = 5)		female(n = 5)		all(n = 10)	
	Mean	SD	Mean	SD	Mean	SD
Kel(1/h)	0.0855	0.0054	0.0930	0.0150	0.0892	0.0113
t_1/2_(h)	8.13	0.50	7.59	1.03	7.86	0.82
t_max_(h)	2.25	1.09	3.40	0.89	2.83	1.12
C_max_(ng/ml)	11.7236	3.9680	11.2417	3.4355	11.4826	3.5083
AUC_0-t_(ng·h/ml)	77.63	18.06	99.48	32.40	88.55	27.28
AUC_0-∞_(ng·h/ml)	78.78	18.38	100.66	32.88	89.72	27.63
Vd(ml)	156,393	42,435	114,794	26,385	135,593	39,880
CL(ml/h)	13,250.6	3023.2	10,611.3	2644.6	11,930.9	3017.5
MRT_0-t_(h)	9.51	1.07	10.28	1.55	9.90	1.32
MRT_0-∞_(h)	10.24	1.23	10.84	1.80	10.54	1.48

All values are mean (±SD)

*Kel* Rate constant for elimination, *t*_*1/2*_ Apparent plasma terminal elimination half-life, *tmax* Time to maximum plasma concentration, *C*_*max*_ Maximum plasma concentration, *AUC*_*0-t*_ AUC from 0 h to time t, *AUC*_*0-∞*_ AUC from time 0 extrapolated to infinity, *Vd* Volume of distribution during terminal elimination phase, *CL* Apparent clearance, *MRT*_*0-t*_ The average residence time of zero time to t, *MRT*_*0-∞*_ The average residence time of zero time to infinite time

**Table 5:** Comparison of pharmacokinetic (PK) parameters in male and female subjects after multipledose of 0.5 mg metolazone tablets

PK parameter	male(n = 5)		female(n = 5)		all(n = 10)	
	Mean	SD	Mean	SD	Mean	SD
Kel(1/h)	0.0871	0.0250	0.1042	0.0186	0.0957	0.0227
t_1/2_(h)	8.41	2.00	6.82	1.19	7.61	1.76
t_max_(h)	1.55	0.41	1.70	0.45	1.63	0.41
C_max_(ng/ml)	7.4484	1.5539	8.9449	2.4602	8.1967	2.0941
AUC_0-t_(ng·h/ml)	50.05	13.97	52.64	17.56	51.35	15.02
AUC_0-∞_(ng·h/ml)	51.18	14.42	53.33	17.74	52.26	15.28
C_min_(ng/ml)	0.4646	0.2596	0.3278	0.1888	0.3962	0.2258
C_avg_(ng/ml)	1.8842	0.4668	2.0349	0.5978	1.9595	0.5118
DF(%)	382.5	80.7	448.7	192.2	415.6	143.3
CL(ml/h)	11,630.9	2958.5	10,903.6	2861.5	11,267.2	2770.6
MRT_0-∞_(h)	9.59	2.23	8.36	1.62	8.97	1.94
Vd(ml)	140,134	46,822	104,116	19,283	122,125	38,729
AUC_ss_(ng·h/ml)	45.22	11.20	48.84	14.35	47.03	12.28
Accumulation	1.17	0.08	1.10	0.04	1.13	0.07
DF/τ	15.9	3.4	18.7	8.0	17.3	6.0

All values are mean (±SD)

*Kel* Rate constant for elimination, *t*_*1/2*_ Apparent plasma terminal elimination half-life, *t*_*max*_ Time to maximum plasma concentration, *C*_*max*_ Maximum plasma concentration, *AUC*_*0-t*_ AUC from 0 h to time t, *AUC*_*0-∞*_ AUC from time 0 extrapolated to infinity, *C*_*min*_ Minimum observed concentration, *C*_*avg*_ Average plasma concentration, *DF* Fluctuation, *CL* Apparent clearance, *MRT*_*0-∞*_ The average residence time of zero time to infinite time, *Vd* Volume of distribution during terminal elimination phase, *AUCss* AUC of distribution at steady state, *DF/τ* Fluctuation

### Effect of food

When metolazone was administered with a high-fat food, t_max_ was prolonged by 100% in contrast to the fasting condition. The total exposure (AUC_0-t_ and AUC_0-∞_) and C_max_ were not influenced by administration under food conditions. The pharmacokinetic parameters for metolazone (1 mg tablet) administration under fasting or fed conditions are shown in Fig. 4. The statistical analysis is presented in Table 6.

**Table 6:** Pharmacokinetic parameters of metolazone after a single oral 1 mg dose: effect of food

	Metolazone(1 mg)	
Parameter	Fasted (*n* = 10)	Fed (*n* = 10)
Kel (1/h) t_max_ (h)	0.0966 ± 0.0123 1.43 ± 0.39	0.0892 ± 0.0113 2.83 ± 1.12
t_1/2_(h)	7.28 ± 0.93	7.86 ± 0.82
C_max_ (ng/mL)	13.8666 ± 4.3227	11.4826 ± 3.5083
AUC_0–t_ (h•ng/mL)	91.31 ± 30.39	88.55 ± 27.28
AUC_0–∞_ (h•ng/mL)	92.41 ± 31.38	89.72 ± 27.63
Vd(ml)	120,081 ± 23,585	135,593 ± 39,880
CL(ml/h)	11,755.6 ± 3252.2	11,930.9 ± 3017.5
MRT_0-t_ (h)	8.80 ± 1.50	9.90 ± 1.32
MRT_0-∞_ (h)	9.31 ± 1.77	10.54 ± 1.48

All values are mean (±SD)

*Kel* Rate constant for elimination, *t*_*1/2*_ Apparent plasma terminal elimination half-life, *t*_*max*_ Time to maximum plasma concentration, *C*_*max*_ Maximum plasma concentration, *AUC*_*0-t*_ AUC from 0 h to time t, *AUC*_*0-∞*_ AUC from time 0 extrapolated to infinity, *Vd* Volume of distribution during terminal elimination phase, *CL* Apparent clearance, *MRT*_*0-t*_ The average residence time of zero time to t, *MRT*_*0-∞*_ The average residence time of zero time to infinite time

### Effect of gender

The pharmacokinetic parameters for metolazone were contrasted between males and females. The metolazone pharmacokinetic parameters were not obviously different in men and women. The pharmacokinetic data for men and women are shown in Tables 2, 3, 4 and 5.

### Tolerability

In our study, all of the volunteers well tolerated not only the single oral doses of 0.5 mg, 1 mg, or 2 mg metolazone tablets but also the multiple oral doses of 0.5 mg metolazone tablets once each day for 6 days. Four volunteers exhibited stuffy and running nose symptoms that were identified as the common cold by researchers. The observed symptoms were considered slight, and one subject’s cold was diagnosed as being unconnected to the research treatment. No serious adverse events were observed or reported during the study. The medical examination, electro-cardiograms, and laboratory examinations did not reveal any considerable medical abnormalities.

### Validation of the LC/MS/MS method

#### Selectivity

The verified LC-MS/MS analysis method proved its suitable selectivity. There were no interferences at the retention times for metolazone (4.72 min) or the IS (5.08 min). No obviously interfering peaks from endogenous components were observed in the blank plasma sample. The LC-MS/MS analysis retention time was 4.72 min for metolazone and 5.08 min for zaleplon (IS), with an analysis run time of 5.5 min. These experimental results support the good specificity and selectivity of this quantification method.

#### Sensitivity, linearity, precision, accuracy, and recovery

The lower limit of quantitation (LLOQ) for the analysis was 0.0198 ng/mL for metolazone, with acceptable accuracy and precision. The linearity tendency was obtained for seven different concentrations of metolazone (0.0198, 0.0395, 0.1976, 0.3952, 3.9520, 9.8800, 35.5680 and 79.0400 ng/mL). The peak area response was linear over the entire concentration range of this study. To acquire the calibration data, every experiment was repeated three times at each concentration on three separate days. The calculated value of coefficient of correlation ‘r^2^’ was 0.998. The accuracy and precision of the method were calculated using three concentrations of metolazone in plasma (0.0494, 1.9760 and 63.2320 ng/mL). The 90% confidence intervals for the intra-run (within 1 day) and inter-run (within 3 days) accuracy were in the ranges of 87.2–110.5% and 91.6–106.1%, respectively. The intra-run and inter-run precision was within the ranges of 2.3–16.1% and 5.7–19.0%, respectively. The extraction recoveries (*n* = 6) of metolazone from human plasma at three different concentration levels of 0.0494, 1.9760 and 63.2320 ng/mL were all >88.3%. The mean extraction recovery (n = 6) of IS was 91.20 ± 6.3%.

#### Stability studies

The results of the short- and long-term stock solution stability were assessed and displayed no obvious deviations from the normal values when kept at 4 °C. The stability was measured by selecting the quality controls to three freeze-thaw cycles and comparing them with newly prepared quality control samples. The mean human plasma concentration of metolazone in the quality control samples did not appear to change during the period under the specified storage conditions. The long-term stability of metolazone in the LQC and HQC samples after 113 days of storage at −20 and −70 °C were also investigated, and the RSD values ranged from 1.0% to 14.2%.

## Discussion

We studied the pharmacokinetic characteristics of metolazone in healthy Chinese subjects. We created and confirmed an LC-MS/MS method to determine the human plasma metolazone levels. The validation results of the analysis method for selectivity, sensitivity, linearity precision, accuracy, recovery and stability guarantee that the analysis method was appropriate to quantify the metolazone levels in human plasma. Our analysis method suggests several advantages, such as a fast and simple extraction process, a short chromatographic analysis time (5.5 min for each sample), and good sensitivity (lower limit of quantitation of 0.0198 ng/mL), which makes the analysis method appropriate for the determination of large sample batches derived from the pharmacokinetic property study of metolazone in human plasma.

In this study, the C_max_ and AUC of metolazone were proportional to the dose after single-dosing with 0.5 mg, 1 mg and 2 mg metolazone in healthy Chinese volunteers, thus indicating linear plasma pharmacokinetic characteristics. This investigation suggested that metolazone is rapidly absorbed after oral administration and attains a maximum plasma concentration in approximately 2.0 h. The elimination half-time of metolazone after a single oral dose in healthy Chinese subjects is approximately 7.5 h (Figs. 2, 3, 4 and 5 and Table 1). The pharmacokinetic results from this investigation are in accordance with those from previously published reports in the article. After a single oral dose of 0.5, 1 and 2 mg metolazone, the t_1/2_ values were 6.6 ± 2.8, 7.9 ± 1.2 and 7.6 ± 1.9 h, respectively [1]. In this investigation, metolazone attained a steady state concentration close to the fourth day during the repeated oral administration stage, which is consistent with the previous literature [2–5].

The compared results of the pharmacokinetic parameters between men and women volunteers suggested no obvious differences between the groups, indicating that gender did not influence the pharmacokinetic characteristics of metolazone. Dose alterations with respect to gender in the Chinese volunteers were not evaluated (Tables 2, 3 and 5).

The study of the food effect on the single oral dose pharmacokinetics of metolazone tablets in Chinese healthy volunteers showed a significant increase in t_max_. The AUC_0-t_, AUC_0-∞_, C_max_, C_avg_, CL, Vd, MRT and other pharmacokinetic parameters were not influenced by high-calorie food intake during the test (Table 6).

The t_max_ of metolazone was 1.43 ± 0.39 h in the fasted state and 2.83 ± 1.12 h in the fed state, suggesting that compared with the fasted state, food prolongs the exposure to metolazone in healthy Chinese volunteers (Fig. 4). Food is recognized to result in clinically obvious variations in the pharmacokinetic properties of numerous drugs. The t_max_ significantly increased, possibly because food delayed the gastric emptying. The absorption time was delayed when metolazone was administered with food. The pharmacokinetic results suggest that metolazone can be taken without considering the effects of food when taken orally in its present dose and formulation.

## Conclusions

Both a single dose and multiple doses of metolazone were demonstrated to yield rapid absorption and continued metolazone concentrations in healthy Chinese volunteers. Linear pharmacokinetic parameters can be applied to depict the pharmacokinetics from the single dose data. We found that the metolazone steady-state concentrations were attained on approximately the fourth day during each day of the multiple dose administration. The results suggested that multiple doses of metolazone did not display different distribution and elimination features compared with single doses and that metolazone did not accumulate after repeated administration. The value of t_max_ significantly increased in the fed state, potentially because food slows the emptying of the stomach. Gender did not seem to influence the pharmacokinetic parameters of metolazone, and all of the metolazone treatments in this study were well tolerated.

## Abbreviations

AUC: Area under the concentration time curve
AUC_0-∞_: AUC from time 0 extrapolated to infinity
AUC_0-t_: AUC from 0 h to time t
AUCss: AUC of distribution at steady state
C_avg_: Average plasma concentration; maximum plasma concentration
CL: Apparent clearance
C_max_: Maximum plasma concentration
C_min_: Minimum observed concentration
DF/τ: Fluctuation
K_el_: Rate constant for elimination
MRT_0-∞_: The average residence time of zero time to infinite time
t_1/2_: Apparent plasma terminal elimination half-life
t_max_: Time to maximum plasma concentration
Vd: Volume of distribution during the terminal elimination phase
Vss: Volume of distribution at steady state
